# Clinical Observations on Postless Fiber-Reinforced Composite Restorations in Endodontically Treated Teeth with Immediate Pre-Endodontic Dentin Sealing and an Internal Adhesive Ferrule: A Case Series

**DOI:** 10.3390/dj14030136

**Published:** 2026-03-02

**Authors:** Alexander Bonchev

**Affiliations:** Department of Conservative Dentistry, Faculty of Dental Medicine, Medical University, 1431 Sofia, Bulgaria; a.bonchev@fdm.mu-sofia.bg

**Keywords:** immediate pre-endodontic dentin sealing, intra-orifice barrier, fiber-reinforced resin composite, internal adhesive ferrule, postless

## Abstract

**Background**: Traditional reliance on intraradicular posts for the restoration of root-filled teeth is decreasing due to advances in adhesive dentistry. Immediate pre-endodontic dentin sealing (IPDS) aims to protect dentin during endodontic procedures and improve adhesive outcomes. For teeth with minimal remaining structure and absent ferrule, internal adhesive ferrule approaches using fiber-reinforced composites or fiber mesh offer an alternative to posts. **Methods**: Four endodontically treated teeth with severely reduced coronal structure were restored using the IPDS protocol, reinforcement with an internal adhesive ferrule ring and fiber composites, and postless adhesive build-ups. Clinical and radiographic assessments were performed up to 2.5 years post-treatment. **Results**: All teeth remained asymptomatic, with stable periodontal and periapical conditions. Restorations maintained structural integrity and favorable adhesive performance. **Conclusions**: Within the limitations of this small case series, the IPDS approach combined with fiber-reinforced postless restorations showed favorable short-term clinical outcomes. Given the small sample size, case heterogeneity, and lack of a control group, these observations should be considered preliminary, and well-designed, long-term controlled studies are required to confirm the durability and broader applicability of this technique.

## 1. Introduction

Restoration of root-filled teeth is a key phase of endodontic treatment, aiming to recover function and aesthetics while conserving remaining tooth structure. Advances in adhesive dentistry, ceramics, and digital technologies have expanded restorative options, improving outcomes but increasing clinical complexity [1,2,3]. The failure of root-filled teeth may be biological, structural, or periodontal, and contemporary research emphasizes overall tooth survival rather than endodontic success alone [2,4,5].

The use of intraradicular posts in endodontically treated teeth continues to be a subject of debate [6]. It is well established that posts primarily function to provide retention for the core material and do not reinforce the root structure or compensate for the absence of an adequate ferrule. With improvements in adhesive restorative materials, the indication for post placement has diminished, and when posts are indicated, the additional removal of radicular dentine should be minimized [2].

Immediate dentin sealing is a technique in which dentin is hybridized before it comes into contact with saliva, blood, impression materials, or temporary cements in indirect restorations, and has been recommended for more than 30 years [7,8,9,10]. This approach has been demonstrated to enhance bond strength [11,12], improve marginal adaptation [7,13,14], and increase the clinical longevity and success of restorations [7,15,16]. Building on this principle, immediate pre-endodontic dentin sealing (IPDS) involves performing dentin hybridization and partial restoration before initiating root canal treatment or retreatment [7,17,18]. The goal of IPDS is to protect the dentin from the potentially detrimental effects of endodontic irrigants and obturation materials, including sodium hypochlorite, EDTA, and root canal sealers [7]. In some cases, deep subgingival margins are challenging to isolate, so they can be elevated as part of IPDS using the deep margin elevation (DME) technique [19]. Performing dentin hybridization prior to endodontic treatment is expected to enhance adhesion and, consequently, improve the long-term prognosis of endodontically treated teeth [7].

Research consistently shows that the presence of an adequate ferrule effect and sufficient remaining cavity walls significantly improves the biomechanical performance and long-term survival of restorations and root-filled teeth [20,21,22]. Although definitions of the appropriate size vary among studies, clinical and laboratory evidence generally supports a minimum ferrule height of approximately 1.5–2.5 mm and a thickness of about 2 mm as key factors associated with improved tooth survival and reduced failure rates [22,23,24,25,26].

An alternative strategy aimed at reinforcing severely compromised tooth structure and creating an adhesive abutment without the use of intraradicular posts is the internal adhesive ferrule concept [27]. This approach is based on structural bonding, also known as immediate dentin sealing (IDS), and has been described as the formation of a reinforcing circumferential ring within the tooth structure [28].

Recent in vitro studies and systematic reviews consistently demonstrate that fiber-reinforced composite resin, including short fiber-reinforced composites (SFRCs), increase the fracture resistance of endodontically treated teeth compared to conventional composite resins. These materials also tend to promote more favorable, restorable failure patterns, which may facilitate future repair if fracture occurs [29,30,31].

Although the restorative approach combining IPDS and fiber-reinforced composite resins has been comprehensively described by Gerdolle and Martin in 2023 [27], there is still a lack of clinical follow-up data evaluating the long-term behavior of endodontically treated teeth restored using this combined strategy. Short-fiber-reinforced composite resins were introduced more than a decade ago. However, despite their promising mechanical properties, clinical evidence supporting their clinical effectiveness or superiority over conventional resin-based composites remains limited and of low certainty [32]. The majority of available studies are in vitro, where experimental conditions differ substantially from the complex biomechanical and biological challenges encountered in the clinical environment [33,34,35].

Clinical studies assessing the performance of fiber-reinforced composites in endodontically treated teeth are even more scarce [36,37,38]. The few published clinical reports have primarily focused on molars restored with fiber-reinforced composites alone, without the application of IPDS or an internal adhesive ferrule concept. To the best of the authors’ knowledge, no published clinical follow-up data evaluating the combined use of IPDS, internal adhesive ferrule, and fiber-reinforced composite restorations exist.

Therefore, the aim of the present small case series is to document the clinical behavior over a 2.5-year follow-up period of endodontically treated premolars and molars restored using a combined approach of IPDS and fiber-reinforced composite resins applied prior to endodontic treatment, thereby addressing an existing gap in the current literature.

## 2. Materials and Methods

### 2.1. Description of the Restoration Technique

**Figure 1:**
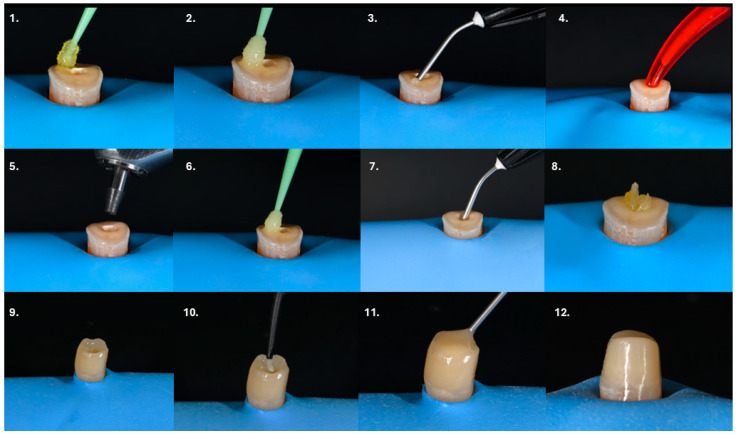
Step-by-step adhesive and restorative protocol. (**1**,**2**) Adhesion achieved using a two-step adhesive system; (**3**) Hybridized dentin covered with a thin layer (≥0.5 mm) of flowable composite resin; (**4**) A GIC base was placed to isolate the sealer from adhesive restorative materials; (**5**) Reactivation of the composite base by airborne particle abrasion with 27-µm aluminum oxide at 2 bar pressure; (**6**) Application of bonding agent (bottle 2) and light curing for 40 s; (**7**,**8**) Optional placement of fiber mesh reinforcement along the cameral dentin walls using bonding agent and flowable composite for adaptation, followed by light polymerization; (**9**) Construction of a circumferential wall of conventional composite resin (composite tube) when no pre-endodontic build-up was present; (**10**) Filling of the composite tube with a short fiber-reinforced composite in a single increment and light curing for 40 s; (**11**) Coverage of the fiber-reinforced composite with conventional composite; (**12**) Final definitive tooth preparation.

The restorative procedures in the presented cases were performed in accordance with the approach proposed by Gerdolle and Martin [27]. After isolation of the operative field with a rubber dam, the thorough removal of demineralized enamel and carious dentin was carried out. Freshly cut dentin was then used for pre-endodontic dentin sealing and, when required, for pre-endodontic build-up. Adhesion was achieved using a two-step adhesive system (Figure 1(1,2)). The hybridized dentin was covered with a thin layer of flowable composite resin with a thickness of at least 0.5 mm (Figure 1(3)). Following completion of endodontic treatment or retreatment, the root canal system was obturated with sealer and gutta-percha. Subsequently, 3 mm of the filling material was removed below the canal orifice level, and a glass ionomer cement (GIC) base was placed to isolate the endodontic sealer from the adhesive restorative materials (Figure 1(4)). The composite base was then reactivated by airborne particle abrasion using 27 µm aluminum oxide at 2 bar pressure (Figure 1(5)). Bonding agent (bottle 2 of the adhesive system) was applied and light-cured for 40 s (Figure 1(6)). Optionally, a fiber mesh reinforcement was placed along the cameral dentin walls. For this step, the fiber mesh was immersed in the bonding agent (bottle 2 of the adhesive system) for 10 min prior to application. A small increment of flowable composite was applied simultaneously with the impregnated fiber mesh to facilitate adaptation and stabilization against the dentinal walls, followed by light polymerization for 40 s (Figure 1(7,8)). Alternatively, the core build-up may be constructed entirely using a short-fiber-reinforced composite. In cases where a pre-endodontic build-up had not been performed, a circumferential wall of conventional composite resin was first constructed, creating a tube-shaped structure (Figure 1(9)). The internal space of this “composite tube” was then filled in a single increment with a short-fiber-reinforced composite and light-cured for 40 s (Figure 1(10)). Care was taken to maintain a 2 mm clearance above the fiber-reinforced composite and cover it with conventional composite (Figure 1(11)). Finally, definitive tooth preparation was completed (Figure 1(12)).

### 2.2. Outcome Assessment

Treatment outcomes were assessed at follow-up according to predefined clinical and radiographic criteria: **1. Patient perception of the treatment outcome** was evaluated based on the presence or absence of spontaneous pain, pain on mastication, sensitivity to percussion or palpation, and functional discomfort during normal chewing; **2. Restorative outcomes** were assessed by clinical examination, focusing on the presence or absence of secondary caries, marginal adaptation, integrity and retention of the restorative material, and, for full-coverage crowns, the marginal fit and continuity of crown margins, cervical adaptation, absence of overcontouring or undercontouring, and integrity of the crown material; **3. Periodontal status** was evaluated by assessing bleeding on probing, tooth mobility, furcation involvement where applicable, and clinical signs of periodontal inflammation; **4. Radiographic outcomes** were assessed using periapical radiographs to evaluate the presence, reduction, or resolution of periapical radiolucencies and to determine the Periapical Index (PAI) score.

## 3. Results

### 3.1. Presentation of Clinical Case #1

#### 3.1.1. Patient Information

A 40-year-old male patient was referred to the endodontic clinic with a chief complaint of mild pain during chewing in the upper left maxillary region. The patient reported that the discomfort had been present for approximately two weeks and occurred exclusively during mastication. No spontaneous pain was noted, and the symptoms did not require the use of analgesic medication. The patient was systemically healthy, with no relevant medical history or known systemic diseases.

#### 3.1.2. Clinical Findings and Diagnosis

**Figure 2:**
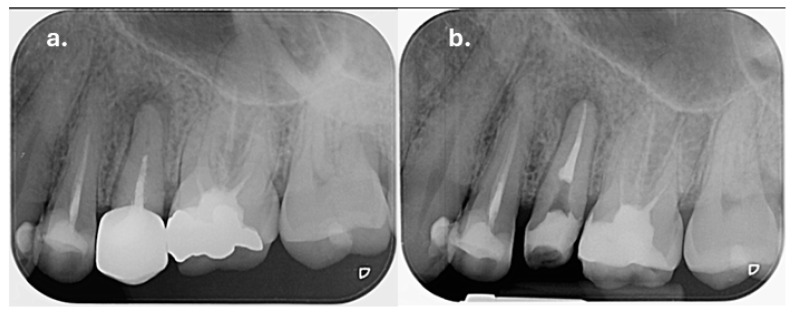
Preoperative radiograph of tooth 25: (**a**) showing the diffuse radiolucency due to the periapical inflammation; (**b**) immediately after root canal obturation.

Clinical examination of the upper left maxillary region revealed extensive restorations on teeth 23, 24, and 26, while tooth 25 was restored with a full-coverage crown. Percussion testing showed tenderness exclusively on tooth 25, whereas adjacent teeth were asymptomatic. Palpation of the buccal mucosa was slightly painful in the area corresponding to the apex of tooth 25. Periapical radiographic examination demonstrated that tooth 25 had been previously endodontically treated and restored with an intraradicular post. A diffuse periapical radiolucency surrounding the root apex was observed, consistent with a Periapical Index (PAI) score of 5, indicating severe apical periodontitis (Figure 2a). In contrast, teeth 24 and 26 also showed evidence of previous root canal treatment but presented no radiographic signs of periapical inflammation. Periodontal evaluation of tooth 25 revealed normal probing depths within physiological limits, with no isolated deep periodontal pockets, and no signs of increased tooth mobility, thereby excluding a primary periodontal lesion. Based on the clinical and radiographic findings, tooth 25 was diagnosed with symptomatic apical periodontitis.

#### 3.1.3. Therapeutic Intervention

Given the extensive coronal damage to tooth 25, the treatment approach prioritized conserving root dentin while re-establishing coronal structure. A radicular post-free strategy was chosen to avoid further weakening the tooth, and immediate pre-endodontic dentin sealing with a layered build-up using the combination of fiber mesh and short fiber-reinforced resin composite was applied to enhance structural support and prepare the tooth for definitive crown restoration.

**First Visit**

After an explanation of the diagnosis and proposed treatment, written informed consent was obtained. Local anesthesia was administered using 1.8 mL of articaine 4% with epinephrine 1:100,000. The existing metal–ceramic crown on tooth 25 was sectioned and removed, followed by rubber dam isolation. Removal of the previous composite restoration revealed secondary caries, which was thoroughly excavated. Clinical examination demonstrated severe coronal destruction, with only the vestibular and mesial dentinal walls remaining supragingivally (approximately 1.5 mm in height), while the palatal and distal walls were absent and extended to the gingival level.

The metallic intraradicular post was removed under copious water cooling using an ultrasonic device (Woodpecker, Guilin, China). Immediate pre-endodontic dentin sealing and build-up were performed in accordance with the protocol described above, using a conventional composite resin (Estelite Asteria, Tokuyama Dental Corporation, Tokyo, Japan).

Endodontic retreatment was then initiated following the identification of a previously untreated canal. The existing root canal filling material was removed, and canal patency was confirmed. After glide path establishment with a size #10 stainless steel K-file, canal shaping was performed using the WaveOne Gold Medium 35/0.06 system (Dentsply Sirona, Ballaigues, Switzerland). Irrigation was carried out with 5.25% sodium hypochlorite activated using a negative-pressure irrigation system (EndoVac, Kerr Corporation, Orange, CA, USA), followed by 17% EDTA and distilled water. The tooth was temporarily sealed.

**Second Visit**

At the one-week follow-up appointment, the patient was asymptomatic, and no exudate was present in the root canal system. After copious final irrigation with activated 5.25% sodium hypochlorite, 17% EDTA, and alcohol, the canals were obturated using a single-cone technique with an epoxy-resin-based sealer (AH Plus, Dentsply Sirona, Konstanz, Germany) (Figure 2b).

**Figure 3:**
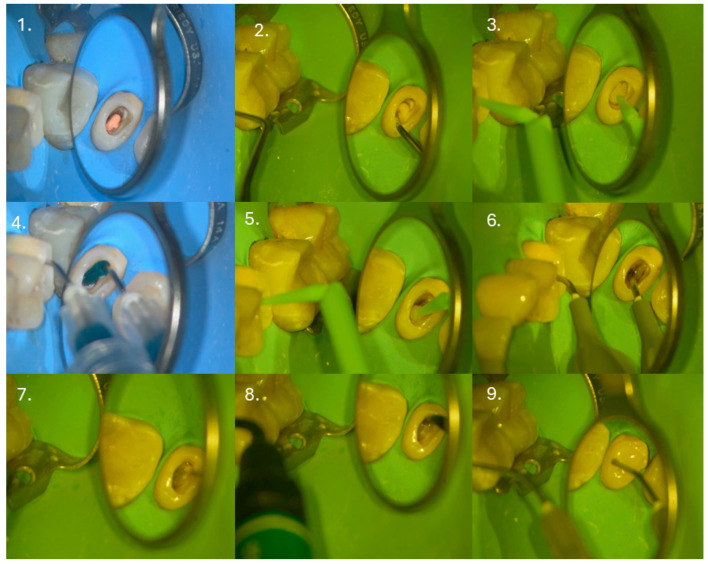
Restorative procedure of the clinical case, illustrating the step-by-step coronal reconstruction following endodontic retreatment and pre-endodontic dentin sealing.

Subsequently, 3–4 mm of the root canal filling was removed apically from the canal orifice (Figure 3(1)), and a glass ionomer cement base (Riva LC HV, SDI, Bayswater, Victoria, Australia) was placed to isolate the sealer (Figure 3(2)) and adapted with a microbrush (Figure 3(3)). After polymerization, the previously placed IPDS was reactivated by airborne particle abrasion using 27 µm aluminum oxide (COXO, Foshan, China), followed by selective 5 s re-etching (Figure 3(4)) and application of the adhesive system (OptiBond FL, Kerr Corporation, Orange, CA, USA) (Figure 3(5)). A thin layer of flowable composite (Filtek Supreme Flowable, 3M Company, St. Paul, MN, USA) was applied (Figure 3(6)), fiber mesh reinforcement (Ribbond^®^, Ribbond Inc., Seattle, WA, USA) was placed and light-cured (Figure 3(7)), and the core build-up was completed with short-fiber-reinforced composite (everX Flow, GC Europe, Leuven, Belgium) (Figure 3(8)). The final 2 mm of the core was restored with conventional composite (Figure 3(9)).

The patient was subsequently referred back to the restorative dentist for definitive crown preparation and prosthetic rehabilitation.

#### 3.1.4. Follow up and Outcomes

**Patient perception:** At the 2.5-year follow-up, the patient reported no spontaneous pain, pain on mastication, sensitivity to percussion or palpation, or functional discomfort.

**Restoration outcome:** Tooth 25 was definitively restored with a metal–ceramic crown. The restoration demonstrated satisfactory marginal adaptation, intact crown material, and adequate retention, with no evidence of secondary caries.

**Periodontal status:** Periodontal examination revealed probing depths within physiological limits, absence of bleeding on probing, and no pathological tooth mobility.

**Figure 4:**
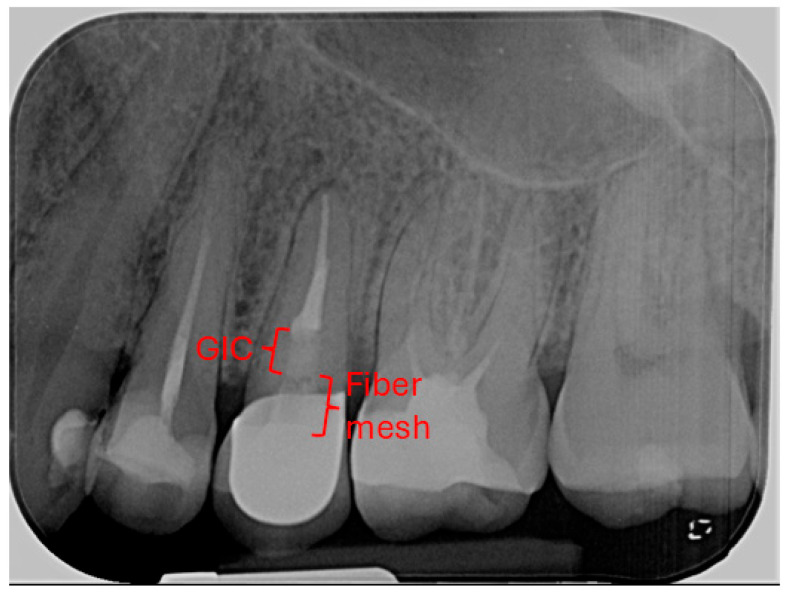
A 2.5-year follow-up periapical radiograph of tooth 25. The GIC base and the fiber mesh component of the coronal restoration are indicated on the radiograph.

**Radiographic signs:** Periapical radiographic assessment showed complete resolution of the previously observed periapical radiolucency, with improvement of the PAI score from 5 at baseline to 1 at follow-up, indicating the re-establishment of normal periapical structures (Figure 4).

After 2.5 years, tooth 25 remained free of symptoms, with radiographs showing the complete resolution of the previous periapical lesion and stable periodontal health. The coronal restoration remained intact, and no complications were noted, indicating that the post-free approach combined with IPDS allowed the tooth to maintain functional and structural stability over the medium-term.

### 3.2. Presentation of Clinical Case #2

#### 3.2.1. Patient Information

A 57-year-old female patient was referred to the endodontic clinic following the incidental radiographic detection of periapical lesions associated with tooth 46 during preoperative assessment for the replacement of an existing metal–ceramic crown. The patient was asymptomatic at the time of presentation. Her medical history was with no reported systemic diseases and no regular medication use. According to the patient’s dental history, the mandibular posterior teeth had undergone endodontic and restorative treatment more than 10 years ago.

#### 3.2.2. Clinical Findings and Diagnosis

**Figure 5:**
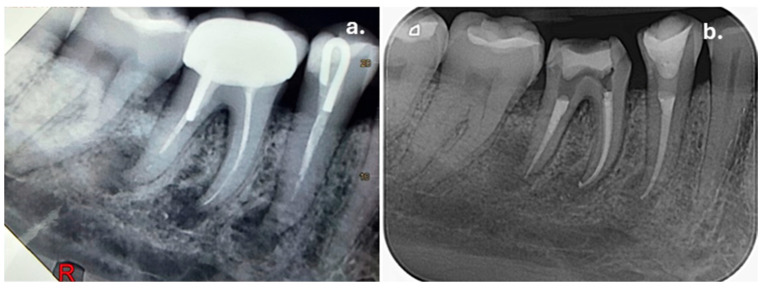
(**a**) Preoperative periapical radiograph of the mandibular right posterior region, showing periapical radiolucencies associated with teeth 45 and 46 prior to endodontic retreatment. The PAI score for both lesions was estimated as 5; (**b**) Post-obturation periapical radiograph of teeth 45 and 46.

Clinical examination revealed that tooth 45 presented with a pre-existing disto-occlusal composite resin restoration, while tooth 46 was restored with a metal–ceramic crown. Both teeth were asymptomatic and did not exhibit sensitivity to percussion or palpation. Periodontal examination demonstrated normal probing depths, with no evidence of periodontal pockets, tooth mobility, or bleeding on probing. Based on the clinical findings and radiographic evidence, both teeth were diagnosed with asymptomatic apical periodontitis (Figure 5a). Endodontic retreatment of teeth 45 and 46 was therefore proposed and discussed with the patient as the treatment of choice.

#### 3.2.3. Therapeutic Intervention

The treatment strategy was designed to prioritize the preservation of radicular dentin while ensuring adequate coronal reinforcement. Considering the substantial loss of coronal structure and the presence of previous restorative procedures, a post-free approach was selected to avoid the additional removal of root dentin and potential weakening of the radicular walls. IPDS combined with subsequent short-fiber-reinforced composite build-up was performed to enhance coronal sealing, improve stress distribution, and provide structural support prior to definitive prosthetic rehabilitation.

**First Visit**

The existing crown on tooth 46 was sectioned and carefully removed. Rubber dam isolation of the entire fourth quadrant was established for both teeth.

**Tooth 45:** The existing composite restoration was removed. A metal wire, presumed to have been placed for restorative retention, was also removed during cavity preparation. Following the removal of carious tissue and confirmation of freshly cut dentin, initial pre-endodontic build-up and IPDS were performed according to the methodology detailed in Materials and Methods and in Clinical Case #1. Root canal debridement and shaping were completed using the WaveOne Gold Primary file (25/07; Dentsply Sirona, Ballaigues, Switzerland). Irrigation was performed with activated 5.25% sodium hypochlorite, 17% EDTA, and distilled water as the final rinse.

**Tooth 46:** The existing composite resin restoration was also removed, revealing a metallic intra-radicular post within the distal root. After careful removal of the surrounding cement, the post was retrieved efficiently using an ultrasonic device under continuous water cooling, minimizing undue stress to the root structure. Residual cement was meticulously eliminated with ultrasonic tips and water irrigation. During this procedure, a non-propagating dentinal crack was identified emanating from the canal orifice. The defect did not extend to the external root surface. The crack was clearly visualized under dental operating microscope magnification without the necessity for dye tests or additional diagnostic adjuncts. Pre-endodontic build-up and IPDS were subsequently completed using the same technique as described for tooth 45. Endodontic retreatment proceeded following the established protocol: the mesial canals were shaped with WaveOne Gold Small (20/07; Dentsply Sirona, Ballaigues, Switzerland) and the distal canal with WaveOne Gold Primary (25/07; Dentsply Sirona, Ballaigues, Switzerland). Irrigation was maintained with activated 5.25% sodium hypochlorite, 17% EDTA, and distilled water, consistent with the protocol applied for tooth 45.

**Second Visit**

At the one-week follow-up appointment, both teeth remained asymptomatic and were suitable for obturation. Root canals were obturated using the single-cone technique with an epoxy-resin-based sealer (AH Plus, Dentsply Sirona, Konstanz, Germany) (Figure 5b). The obturation was intentionally terminated 3 mm apically to the level of the canal orifices to allow space for coronal reinforcement.

Following obturation, the coronal restoration was completed according to the methodology described in Section 2.1, using a short-fiber-reinforced composite for coronal reinforcement, without the use of fiber mesh reinforcement. Upon completion of the endodontic and restorative procedures, the patient was referred back to the restorative dentist for definitive prosthetic rehabilitation.

#### 3.2.4. Follow up and Outcomes

**Patient perception:** At the 2.5-year follow-up, both teeth were asymptomatic, with no spontaneous pain, pain on mastication, sensitivity to percussion or palpation, or functional discomfort.

**Restoration outcome:** Definitive restorative treatment consisted of a metal–ceramic crown for tooth 46 and a composite onlay for tooth 45. The crown on tooth 46 exhibited adequate marginal fit, cervical adaptation, and material integrity. The composite onlay on tooth 45 showed insufficient coverage of the gingival floor, and the patient was referred for corrective restorative management. No secondary caries or loss of restoration retention was detected.

**Periodontal status:** Clinical evaluation demonstrated healthy periodontal conditions for both teeth, characterized by normal probing depths, absence of bleeding on probing, and no pathological mobility.

**Figure 6:**
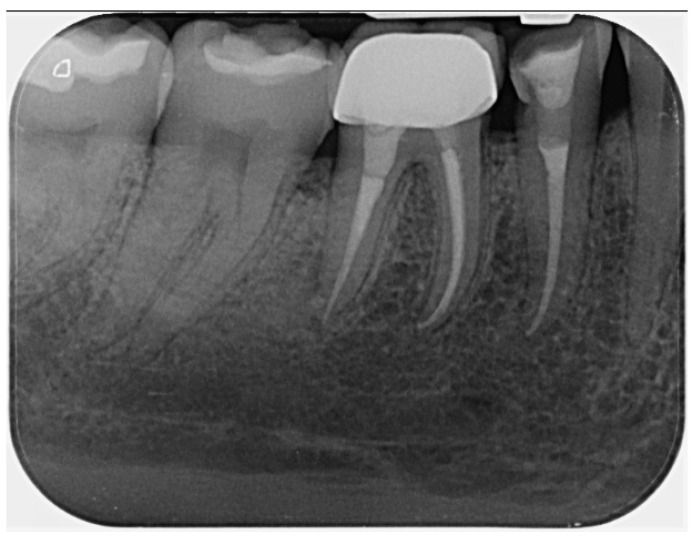
Periapical radiograph obtained at the 2.5-year follow-up, showing teeth 45 and 46 in function with complete resolution of the periapical lesions (PAI score 1).

**Radiographic signs:** At 6 months, radiographs showed a gradual reduction of periapical radiolucencies associated with both teeth. At the 2.5-year follow-up, complete periapical healing was observed, with PAI scores of 1 for both teeth (Figure 6).

At the 2.5-year follow-up, the teeth remained asymptomatic and demonstrated complete periapical healing, stable periodontal conditions, and the maintenance of coronal structural integrity without the use of a radicular post. During this observation period, no signs of propagation of the previously identified dentinal crack on tooth 46 were detected, suggesting that the post-free restorative approach used in this case was compatible with medium-term structural stability.

### 3.3. Presentation of Clinical Case #3

#### 3.3.1. Patient Information

A 47-year-old female patient was referred to the endodontic clinic for a retreatment of tooth 47 as part of a comprehensive oral rehabilitation planned by a prosthetic dentist. The referral was based on prosthetic considerations rather than patient-reported symptoms. At the time of presentation, the patient reported no pain, discomfort, or other complaints related to the involved tooth. The patient’s medical history was noncontributory. She reported being in good general health, with no known systemic diseases and no regular use of medications.

#### 3.3.2. Clinical Findings and Diagnosis

**Figure 7:**
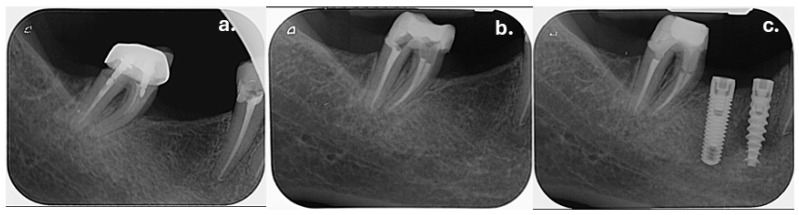
(**a**) Preoperative periapical radiograph of tooth 47, showing previous endodontic treatment, the presence of two intraradicular posts, and marginal discrepancies at the mesial and distal aspects of the metal–ceramic crown; (**b**) Postoperative periapical radiograph following endodontic retreatment and obturation using the single-cone technique; (**c**) Final coronal restoration with a short-fiber-reinforced composite placed as a core build-up material and covered with a conventional composite resin.

Clinical examination revealed that tooth 47 was restored with a metal–ceramic crown. Marginal gaps were detected on both the mesial and distal aspects of the crown during the probing. The probed tooth structure in these areas exhibited a soft consistency, suggestive of secondary caries presence. Percussion and palpation tests were negative. Periodontal examination demonstrated a Grade I furcation involvement, accessed from the lingual aspect, according to the classification proposed by Glickman [31]. No bleeding on probing was observed, and the tooth exhibited no pathological mobility. Radiographic examination revealed that tooth 47 had been previously endodontically treated. Radiolucent gaps were evident at the mesial and distal crown margins (Figure 7a). Additionally, two intraradicular posts were present within the root canals. The periapical tissues appeared normal, with no signs of periapical pathology. Based on the clinical and radiographic findings, the diagnosis was a previously treated tooth with normal apical tissues. Endodontic retreatment was indicated due to coronal microleakage and the associated risk of bacterial contamination of the existing root canal filling material.

#### 3.3.3. Therapeutic Intervention

The treatment plan for this case focused on managing the extensive loss of coronal tooth structure in a tooth with two intraradicular posts while minimizing further weakening of the roots. A post-free approach was selected, combined with IPDS, to preserve radicular dentin and ensure a stable foundation for restoration. The plan included using the space previously occupied by the posts for the placement of short-fiber-reinforced composite resin to reinforce the dentinal structure, while leaving out fiber mesh reinforcement due to the narrow root canal anatomy.

The treatment protocol followed the same sequence described in the previous cases. The existing metal–ceramic crown on tooth 47 was sectioned and removed, followed by the removal of carious dentin and intraradicular posts using an ultrasonic device. After restoration and post removal, only the mesial coronal tooth structure remained, with buccal, lingual, and distal walls at the juxtagingival level. An IPDS and pre-endodontic build-up were performed prior to single-visit endodontic retreatment and obturation using a single-cone technique (Figure 7b). The patient remained asymptomatic at the one-week follow-up. Final coronal restoration was completed as described in Clinical Case #2, using a short-fiber-reinforced composite (everX Flow, GC, Tokyo, Japan) placed to a depth of 3 mm below the canal orifices and subsequently covered with a conventional composite resin (Estelite Asteria, Tokuyama Dental, Tokyo, Japan) (Figure 7c).

After completion of the treatment, the patient was referred back to the prosthetic dentist to continue with the planned full oral rehabilitation.

#### 3.3.4. Follow up and Outcomes

**Patient perception:** At the 2.5-year follow-up, the patient reported no symptoms, including absence of pain or functional discomfort.

**Restoration outcome:** Tooth 47 was definitively restored with a full-coverage crown and functioned as a natural abutment within a fixed prosthetic construction connected to adjacent implant-supported restorations. The crown demonstrated satisfactory marginal fit, cervical adaptation, and material integrity.

**Periodontal status:** Periodontal examination revealed a stable Grade I furcation involvement, unchanged from baseline, with no bleeding on probing and no pathological tooth mobility.

**Figure 8:**
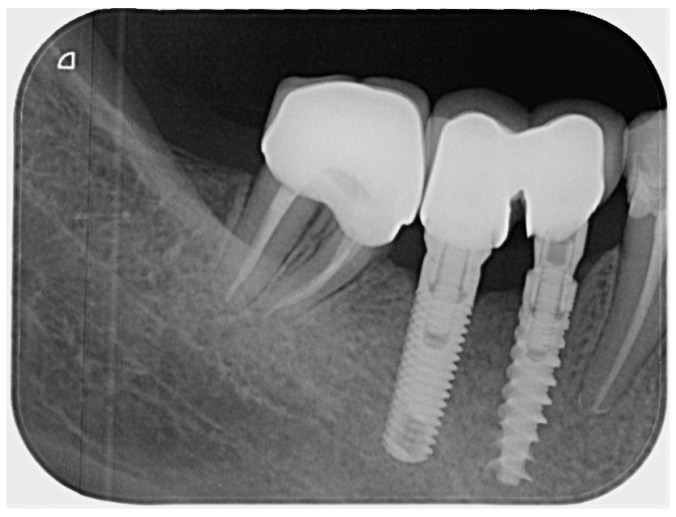
Control periapical radiograph of tooth 47 taken 2.5 years after completion of endodontic and prosthetic treatment.

**Radiographic signs:** Periapical radiographic findings remained stable, with no evidence of new or progressive periapical pathology (Figure 8).

At the 2.5-year follow-up, the tooth remained symptom-free, and the coronal restoration maintained its integrity and fit within the prosthetic framework. Periodontal and radiographic evaluations showed stable conditions, with no new pathology observed, indicating that the selected post-free, IPDS-based restorative approach supported the medium-term maintenance of both function and structure in this case.

### 3.4. Presentation of Clinical Case #4

#### 3.4.1. Patient Information

A 26-year-old male patient presented to the dental clinic with the chief complaint of a fractured restoration at tooth 45. The patient had previously attended a general dental practice one week after the fallen restoration, where the affected tooth was temporized. At the time of presentation, the patient reported no pain or sensitivity associated with the tooth. The patient’s medical history was non-contributory, with no reported systemic diseases, ongoing medical conditions, or regular medication use.

#### 3.4.2. Clinical Findings and Diagnosis

**Figure 9:**
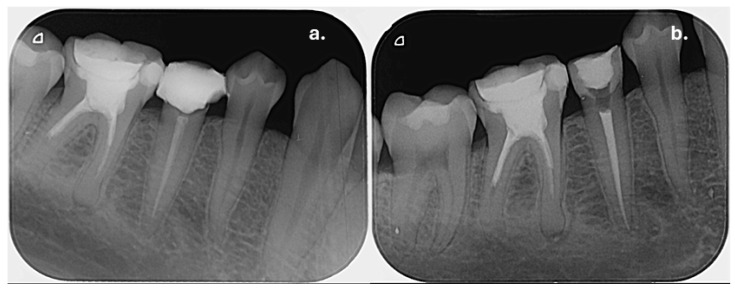
(**a**) Diagnostic periapical radiograph of tooth 45; (**b**) Periapical radiograph following the root canal treatment of tooth 45.

Clinical examination revealed that tooth was 45 restored with a temporary filling material. The tooth was asymptomatic, with negative responses to both percussion and palpation tests. Periodontal probing depths were within normal physiological limits, with no signs of periodontal pathology. Periapical radiographic examination demonstrated a previously endodontically treated tooth with normal periapical tissues (Figure 9a). However, due to prolonged exposure of the root canal filling material to the oral environment for more than one week, endodontic retreatment was indicated, as such exposure may result in bacterial contamination of the root canal system and degradation of the sealing properties of the endodontic sealer.

#### 3.4.3. Therapeutic Intervention

The treatment plan for this case focused on restoring a severely compromised coronal structure in tooth 45 while preserving radicular dentin and avoiding posts. Because the intracanal space was wider, a combination of short-fiber-reinforced composite and fiber mesh reinforcement was planned to maximize structural support and improve the distribution of functional stresses within the remaining tooth tissue. Immediate pre-endodontic dentin sealing and a layered core build-up were incorporated to stabilize the coronal structure prior to obturation, providing a durable foundation for the eventual full-coverage crown.

Following removal of the temporary restoration and thorough caries excavation, clinical evaluation revealed a severely reduced coronal tooth structure at the gingival margin, with no remaining ferrule. The protocol for pre-endodontic build-up and IPDS was performed as previously described in earlier cases. Endodontic retreatment was then carried out according to the established protocol. At the same visit, the root canal system was obturated using a single-cone technique with an epoxy-resin-based sealer (Figure 9b).

**Figure 10:**
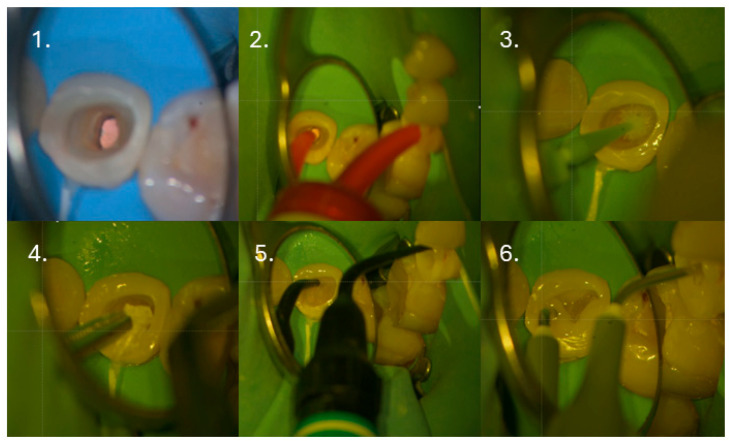
Clinical sequence of the postless restorative approach using fiber mesh reinforcement prior to final crown placement: (**1**). Removal of gutta-percha to 3 mm below the level of the orifice; (**2**). Placement of GIC barrier; (**3**). Re-application of the bonding agent; (**4**). Adaptation of the fiber mesh to the cavity walls; (**5**). Placement of the flowable short-fiber-reinforced resin composite; (**6**). Final layer of the core build-up using flowable resin composite.

One week after completion of the endodontic procedure, the tooth was restored using a postless approach with fiber mesh reinforcement, following the protocol and materials described in Clinical Case #1 (Figure 10).

The patient was subsequently referred for full crown preparation and definitive prosthetic restoration.

#### 3.4.4. Follow up and Outcomes

**Patient perception:** At the 2.5-year follow-up, the tooth remained asymptomatic, with no spontaneous pain, pain on mastication, sensitivity to percussion or palpation, or functional discomfort.

**Restoration outcome:** The tooth had received a postless restoration but had not yet been definitively restored with a full-coverage crown. Despite the absence of definitive prosthetic coverage, no signs of restorative failure were observed. Definitive crown placement was recommended to ensure long-term structural protection.

**Periodontal status:** Clinical examination revealed no signs of periodontal inflammation, bleeding on probing, or pathological mobility.

**Figure 11:**
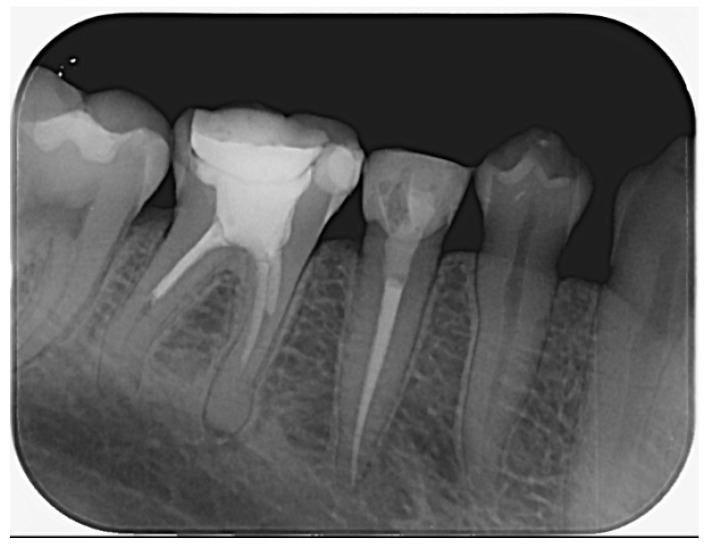
Clinical and radiographic follow-up of tooth 45 at 2.5 years post-endodontic retreatment and postless restoration.

**Radiographic signs:** Periapical radiographic evaluation demonstrated stable periapical conditions, with no evidence of pathological changes (Figure 11).

At the 2.5-year follow-up, the tooth remained symptom-free, with stable periodontal conditions and no functional discomfort. The postless restoration, combining short-fiber composite resin and fiber mesh, maintained coronal integrity despite the absence of definitive crown coverage for a medium-term follow-up period.

## 4. Discussion

Due to the advancements in adhesive procedures achieved over recent decades, the use of radicular posts has been called into question [6]. Their routine use in the restoration of endodontically treated teeth remains controversial among dental researchers and clinicians [39]. It should be noted that the primary function of intraradicular posts is to provide retention for the core restoration rather than to increase the fracture resistance of the root [39]. A major concern associated with post placement is the increased risk of non-repairable root fractures [39,40,41]. Several studies have demonstrated that post placement does not significantly improve restoration outcomes in posterior teeth lacking a ferrule or in anterior teeth with an adequate ferrule [42,43,44,45].

In this context, the choice of core build-up material may also influence clinical outcomes. The use of bulk-fill composites may reduce the occurrence of voids and interlayer defects, while short-fiber-reinforced composites have been proposed to function as load-bearing barriers, potentially enhancing stress distribution within the restored tooth [39].

The literature indicates that fiber-reinforced composites constitute a conservative, cost-effective, and time-efficient biomimetic strategy that enhances physical properties, reduces polymerization shrinkage stress, and contributes to fracture prevention in extensive restorations [46,47]. Structurally composed of a polymer matrix, reinforcing fibers, and their interface, these systems improve mechanical behavior by transferring functional stresses from the resin matrix to the fibers, thereby reinforcing weakened tooth structures and lowering fracture risk [48]. Their reinforcing efficacy is influenced by variables such as fiber type, orientation, quantity, impregnation, and adhesion to the resin matrix, with fiberglass and polyethylene fibers being the most commonly used due to their favorable mechanical and esthetic characteristics [49,50,51]. Nevertheless, despite numerous predominantly in vitro studies evaluating the fracture resistance of fiber-reinforced composite restorations, the available evidence remains inconsistent and controversial [52,53,54,55].

Within this framework, the present case reports add medium-term clinical evidence on the performance of this restorative approach, with a 2.5-year follow-up in a field where robust clinical data are still limited. However, the positive outcomes cannot be attributed exclusively to the use of fiber-reinforced composites, as several confounding clinical and procedural factors may have contributed to the results.

One of the specific factors warranting discussion is the immediate preendodontic dentin sealing (IPDS) procedure, which was applied in all presented cases. Sodium hypochlorite (NaOCl), commonly employed as an endodontic irrigant, acts as a potent oxidizing agent that leaves an oxygen-rich layer on the dentinal surface [56]. This residual oxygen hampers resin polymerization, increases microleakage, and compromises the bond strength of adhesive systems to root canal dentin [57,58,59]. As a result, NaOCl negatively affects the integrity of the resin–dentin interface, leading to diminished adhesive performance [60]. The application of IPDS has been shown to mitigate these negative effects of NaOCl, thereby enhancing the quality and durability of adhesive bonding [27]. Another factor that may influence adhesive effectiveness is the type of endodontic sealer used, as its chemical composition plays a critical role in subsequent restorative procedures [61,62]. Eugenol-based sealers are known to impair the polymerization of resin-based materials due to the inhibitory action of eugenol, leading to significantly lower bond strengths when compared with epoxy-resin-based sealers [61,62]. In contrast, epoxy-resin-based sealers are generally associated with superior bond strength and are therefore preferred when resin composite core build-ups or fiber post cementation are anticipated [63]. This consideration underpinned the selection of epoxy-resin-based sealers in the present cases.

Carvalho et al. [7] reported that, in the absence of IPDS, the repreparation of approximately 0.5–1.0 mm of dentin may be required to remove tissue infiltrated or impregnated with endodontic sealer, thereby exposing a substrate suitable for adhesive procedures [64,65]. However, further investigations are necessary to determine the precise amount of dentin that must be removed to reliably achieve optimal bonding conditions [7]. The need for such repreparation can be avoided through the use of IPDS, which is particularly advantageous in severely compromised teeth. Moreover, IPDS offers an additional benefit by contributing to the reinforcement of the remaining tooth structure [7], which is an important consideration in the present cases, which were managed using a multi-visit treatment approach. Another advantage of this technique is the ability to perform deep margin elevation (DME), which was required in the presented cases #1, #3 and #4 to facilitate adequate isolation and optimize adhesive and restorative procedures [19].

One of the key strategies for minimizing the effects of tensile and bending stresses is the placement of reinforcing fibers in close proximity to the dentinal walls, where the highest tensile, bending, and polymerization shrinkage stresses are concentrated [27]. This concept has traditionally justified the use of fiber posts, which are intended to provide both retention and stress absorption. However, a considerable number of failures have been reported in crowns supported by fiber post-retained abutments, indicating that fulfilling these combined mechanical demands may be challenging in routine clinical practice [40,66,67,68].

To address these limitations, the use of short-fiber-reinforced composite resins and/or fiber meshes in combination with conventional composite materials has been proposed [27,69,70,71,72,73]. Increasing the fiber content within the composite and enabling their strategic placement adjacent to dentinal walls have been identified as promising alternatives to conventional post placement [45,54,74]. This approach enhances the overall mechanical performance of the abutment and contributes to improved bond strength by partially absorbing polymerization shrinkage stresses [73,74]. Despite these positive features, which have been largely demonstrated in laboratory studies, recent clinical investigations have shown no statistically significant difference between fiber-reinforced composites and conventional composites for the restoration of endodontically treated teeth [36,37]. A key limitation of the present case series is the absence of a control group which precludes the direct comparison of outcomes. Furthermore, the small sample size and heterogeneity of the included cases, along with procedural variations, may have influenced the observed results. Nevertheless, the primary aim of this report is to illustrate the clinical behavior of teeth restored using this approach and to emphasize the need for long-term, well-designed, controlled, randomized clinical trials. It is also noteworthy that premolars were included in this series, whereas most previous clinical trials have focused exclusively on endodontically treated molars [36,37,38]

Although the ESE suggests that post placement may be beneficial, but not mandatory, in teeth with no remaining coronal dentin walls, particularly in anterior teeth and premolars [2], a postless restorative approach was chosen in the present cases after thorough discussion with the patient. Importantly, the treated teeth exhibited a reduced, yet still existing, amount of coronal tooth structure. In accordance with ESE guidelines, additional mechanical dentin removal for post placement should be avoided whenever possible [2]. Consequently, the selected restorative strategy is justified, as it preserves sound tooth structure and eliminates the need for post space preparation. When the coronal portion of the root canal is narrow and adaptation of a fiber mesh is not feasible, the restoration can be completed using a short-fiber-reinforced composite as the central component of the core build-up [27], as demonstrated in cases #2 and #3.

In clinical case #2, a dentinal crack was identified on the distal aspect of the root, originating from the root canal space but not extending to the external root surface. This defect was presumed to be a consequence of the previous endodontic treatment or, more likely, related to earlier post cementation. In this case, the post had been cemented in a non-axial orientation relative to the long axis of the tooth, which may have contributed to stress concentration within the root dentin. Dentinal cracks following post placement are primarily attributed to mechanical stresses generated during post space preparation and post cementation [75,76]. The magnitude of vertical load and torsional forces applied during post space preparation varies depending on the drill system used, with higher forces being associated with an increased risk of microcrack formation [75]. Furthermore, post cementation, particularly when excessive seating pressure is applied, may induce transient deformation of the root, thereby contributing to crack initiation or propagation [77]. Additional predisposing factors include dentin dehydration, which can independently promote crack formation irrespective of instrumentation, as well as pre-existing structural defects resulting from prior restorative or endodontic procedures [78]. Post-removal techniques represent another potential risk factor, as they involve further mechanical manipulation of the root structure [79]. Radiographic signs of complicated dentinal crack or root fracture typically include J-shaped or angular osseous defects, which may be more readily detected using cone-beam computed tomography (CBCT). Clinically, dentinal cracks or root fractures may present with pain on biting or upon release of pressure, and in more advanced cases, with deep, isolated periodontal probing depths localized to the area adjacent to the fracture [80,81]. At the 2.5-year follow-up, no radiographic or clinical signs of crack propagation were observed. Despite methodological heterogeneity among available studies, recent evidence suggests that teeth with reduced coronal structure or mild to moderate dentinal cracks may still demonstrate favorable medium-term survival outcomes [25,82]. Nevertheless, it is difficult to attribute the favorable outcome exclusively to the restorative technique involving IPDS and fiber-reinforced composite, as the tooth was definitively restored with a crown immediately after completion of the endodontic treatment, in accordance with recommendations in the literature [2,83]. Moreover, to the best of our knowledge, there are currently no clinical studies that longitudinally evaluate endodontically treated teeth with dentinal cracks restored using fiber-reinforced composite materials.

Another important aspect that warrants discussion is the material used to provide isolation between the root canal filling material and the coronal composite resin restoration. According to the technique proposed by Gerdolle and Martin [27], the placement of a glass-ionomer cement as an intraorifice barrier (IOB) is recommended to prevent potential interactions between adhesive systems and endodontic sealers. The literature indicates that GICs possess many of the ideal characteristics originally proposed for intraorifice barrier materials [84,85]. These include self-adhesive properties with satisfactory chemical bonding to root dentin [86], favorable biocompatibility, a coefficient of thermal expansion similar to that of dental tissues, and antibacterial activity, primarily attributed to their low initial pH and sustained fluoride release [85,87]. The use of GIC is particularly advantageous when resin-based composites are employed coronally, as many adhesive systems contain acetone as a solvent [85]. Previous studies have demonstrated that acetone-based adhesives exhibit compromised polymerization when applied directly over gutta-percha, likely due to the leaching of gutta-percha components that interfere with the polymerization process [88,89]. By acting as a physical and chemical barrier, GIC can effectively minimize this adverse interaction. Nevertheless, one limitation of GIC application should be considered: in narrow canal spaces, placement may be technically challenging and associated with an increased risk of void formation. To overcome this limitation, the use of encapsulated GIC for direct application, as performed in case #4, may improve handling and adaptation.

Alternatively, the literature suggests that flowable resin composites may also be suitable materials for intraorifice barriers due to their superior adaptation to internal dentinal walls [85]. However, their reduced filler content, which confers low viscosity, is associated with increased polymerization shrinkage compared with conventional resin-based composites [90]. In cases #2 and #3, due to the narrow canal anatomy and the need to enhance adaptation, a thin layer of flowable composite was therefore used as an interfacial layer between the fiber-reinforced composite and the gutta-percha.

### 4.1. Limitations

The present study has several limitations that should be acknowledged. First, the findings are based on a series of case reports, which inherently lack statistical power and do not allow for generalization of the results. Additionally, clearly defined inclusion and exclusion criteria were not established for participant selection. The cases involved different teeth with varying diagnoses and degrees of remaining tooth structure, introducing heterogeneity that may have influenced the outcomes. Furthermore, the clinical protocol of the technique was slightly modified in accordance with the specific requirements of each individual case, which limits standardization and makes direct comparison between cases more difficult. The proposed approach should be interpreted as reflecting the author’s clinical perspective and experience rather than serving as a standardized treatment guideline.

### 4.2. Clinical Significance and Future Perspectives

Most studies addressing post-endodontic restorations using fiber-reinforced composite materials are limited to in vitro investigations, while clinical evidence remains scarce. To the best of the author’s knowledge, the present case follow-ups of up to 2.5 years represent the first documented clinical outcomes related to a restorative technique combining the PIDS approach with short-fiber-reinforced resin composites and fiber mesh build-ups. These preliminary clinical observations suggest that this approach may represent a viable restorative option for structurally compromised endodontically treated teeth. However, the results should be interpreted with caution. Longer follow-up periods are required to assess long-term performance and potential failure modes. Furthermore, well-designed randomized clinical trials with standardized protocols are necessary to provide robust evidence and to further clarify the clinical effectiveness of this technique within the ongoing and controversial debate surrounding post-endodontic restorative strategies.

## 5. Conclusions

Within the limitations of this case series, the 2.5-year clinical and radiographic follow-ups of the endodontically treated teeth presented here, restored using the IPDS approach combined with fiber-reinforced resin composite build-ups, showed favorable outcomes in these individual cases. These findings are strictly descriptive of the cases reported and should not be generalized beyond this series. The observations provide clinical insight into treatment planning and medium-term outcomes, but further well-designed, controlled, and long-term studies are needed to evaluate the predictability and durability of this restorative strategy.

## Data Availability

The original contributions presented in this study are included in the article. Further inquiries can be directed to the corresponding author.

## Abbreviations

The following abbreviations are used in this manuscript:

ESE	European Society of Endodontology
IPDS	Immediate Pre-endodontic Dentin Sealing
IOB	Intra-orifice barrier
DME	Deep Margin Elevation
